# Telemonitoring Versus Usual Care for Elderly Patients With Heart Failure Discharged From the Hospital in the United States: Cost-Effectiveness Analysis

**DOI:** 10.2196/17846

**Published:** 2020-07-06

**Authors:** Xinchan Jiang, Jiaqi Yao, Joyce HS You

**Affiliations:** 1 School of Pharmacy Faculty of Medicine The Chinese University of Hong Kong Shatin, NT China (Hong Kong)

**Keywords:** telemedicine, heart failure, hospitalization, cost, quality-adjusted life year, cost-effectiveness analysis

## Abstract

**Background:**

Telemonitoring-guided interventional management reduces the need for hospitalization and mortality of patients with chronic heart failure (CHF).

**Objective:**

This study aimed to analyze the cost-effectiveness of usual care with and without telemonitoring-guided management in patients with CHF discharged from the hospital, from the perspective of US health care providers.

**Methods:**

A lifelong Markov model was designed to estimate outcomes of (1) usual care alone for all postdischarge patients with CHF (New York Heart Association [NYHA] class I-IV), (2) usual care and telemonitoring for all postdischarge patients with CHF, (3) usual care for all postdischarge patients with CHF and telemonitoring for patients with NYHA class III to IV, and (4) usual care for all postdischarge patients with CHF plus telemonitoring for patients with NYHA class II to IV. Model inputs were derived from the literature and public data. Sensitivity analyses were conducted to assess the robustness of model. The primary outcomes were total direct medical cost, quality-adjusted life years (QALYs), and incremental cost-effectiveness ratio (ICER).

**Results:**

In the base case analysis, universal telemonitoring group gained the highest QALYs (6.2967 QALYs), followed by the telemonitoring for NYHA class II to IV group (6.2960 QALYs), the telemonitoring for NYHA class III to IV group (6.2450 QALYs), and the universal usual care group (6.1530 QALYs). ICERs of the telemonitoring for NYHA class III to IV group (US $35,393 per QALY) and the telemonitoring for NYHA class II to IV group (US $38,261 per QALY) were lower than the ICER of the universal telemonitoring group (US $100,458 per QALY). One-way sensitivity analysis identified five critical parameters: odds ratio of hospitalization for telemonitoring versus usual care, hazard ratio of all-cause mortality for telemonitoring versus usual care, CHF hospitalization cost and monthly outpatient costs for NYHA class I, and CHF hospitalization cost for NYHA class II. In probabilistic sensitivity analysis, probabilities of the universal telemonitoring, telemonitoring for NYHA class II to IV, telemonitoring for NYHA class III to IV, and universal usual care groups to be accepted as cost-effective at US $50,000 per QALY were 2.76%, 76.31%, 18.6%, and 2.33%, respectively.

**Conclusions:**

Usual care for all discharged patients with CHF plus telemonitoring-guided management for NYHA class II to IV patients appears to be the preferred cost-effective strategy.

## Introduction

### Background

Chronic heart failure (CHF) is a condition that imposes a major burden on health care systems and the society. Globally, it is estimated that 37.3 million individuals suffer from CHF, and the prevalence continues to rise [1]. In the United States, the number of patients with CHF was nearly 6.2 million in 2016 and is projected to be over 8 million in the year 2030 [2]. CHF is the most common reason for admission, and hospitalization for CHF is highly associated with readmission within 30 days (20%-30%) and 6 months (50%) [3]. The total cost of care for CHF is expected to rise from US $30.7 billion in 2012 to US $69.8 billion in 2030 [4].

The use of digital health interventions in the provision of remote health care services is a promising strategy to improve the clinical outcomes of CHF. Telemonitoring allows remote daily monitoring of patients’ vital signs and, therefore, enables detection of clinical deterioration and early clinical interventions. The Efficacy of Telemedical Interventional Management in Patients with Heart Failure II (TIM-HF2) study compared the efficacy of the telemedical interventional management for CHF patients (New York Heart Association [NYHA] class II or III) along with usual care versus usual care only [5]. It was reported that the structured telemonitoring-based management reduced the percentage of days lost to unplanned cardiovascular admission and all-cause death (4.88% vs 6.64%; *P*=.05) and lowered the all-cause mortality rate (7.86% vs 11.34%; *P*=.03).

Telemonitoring also engages patients in CHF self-care and improves the quality of patients’ self-management at home. In the Trans-European Network–Home-Care Management System study, the telemonitoring intervention consisted of twice-daily patient self-management and monitoring of weight, blood pressure, heart rate, and rhythm with automated devices linked to a cardiology center [6]. The study found that the mean duration of admissions was reduced by 6 days (95% CI 1-11 days) in the home telemonitoring group versus the usual care group. One-year mortality was higher in the usual care group (45% vs 29%; *P*=.03).

### Objectives

Clinical findings have indicated telemonitoring to be beneficial to patients with CHF, yet few studies examined the cost-effectiveness of telemonitoring on the management of CHF [7]. The aim of this study was to analyze the cost-effectiveness of usual outpatient care with and without telemonitoring for patients with CHF who were discharged from the hospital, from the perspective of the US health care providers.

## Methods

### Study Design

**Figure 1:**
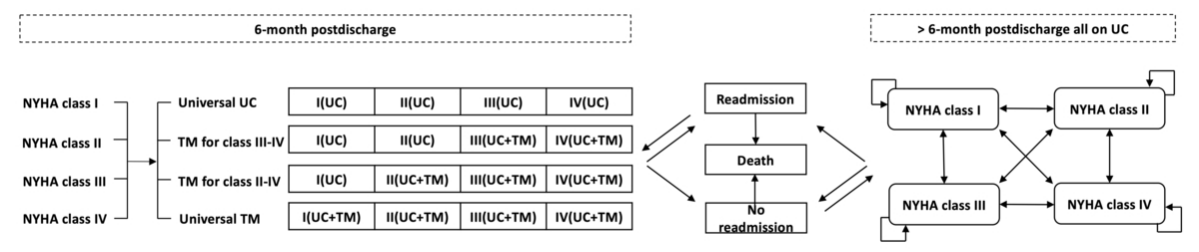
Simplified model structure. NYHA: New York Heart Association; TM: telemonitoring; UC: usual care; universal UC: UC only for all patients; TM for class III to IV: UC for all patients and TM for NYHA class III to IV patients; TM for class II to IV: UC for all patients and TM for NYHA class II to IV patients; universal TM: UC plus TM for all patients.

A lifelong Markov model was constructed using TreeAge Pro 2019 (TreeAge Software Inc) to project the long-term clinical and economic outcomes of a hypothetical cohort of 65-year-old patients with CHF who were recently discharged for CHF-related hospitalization (Figure 1) in the US health care setting. The Markov model is an analytical framework in which the hypothetical cohort of patients transits between the mutually exclusive health states, with costs and health outcomes aggregated over successive cycles. The following strategies were examined in the present model: (1) universal usual care only for all patients (NYHA class I-IV), (2) universal usual care for all patients and telemonitoring for NYHA class III to IV patients (telemonitoring for class III-IV group), (3) universal usual care for all patients and telemonitoring for NYHA class II to IV patients (telemonitoring for class II-IV group), and (4) universal usual care plus telemonitoring for all patients (universal telemonitoring group). The model time horizon was 35 years with 6-month cycles for lifelong simulation of 100 years of age maximum. The primary outcome measures were direct medical costs, quality-adjusted life years (QALYs), and incremental cost-effectiveness ratio (ICER) as additional cost per QALY gained.

As reported by the ancillary Digitalis Investigation Group (DIG) trial, a retrospective cohort study of 988 patients with NYHA class I to IV heart failure, the NYHA classification was associated with the risk of hospitalization and mortality [8]. In this model, we stratified the hypothetical cohort of recently discharged patients with CHF by the NYHA classification. Subjects of all study arms entered the present model at the Markov status *NYHA class I*, *NYHA class II*, *NYHA class III*, or *NYHA class IV*. In each 6-month cycle, all patients might be readmitted for CHF-related hospitalization and might experience all-cause death. For those who survived at the end of cycle, they might remain in the same NYHA classification or transit (improve or progress) to another NYHA classification.

Patients in all study arms received usual care: physician’s office visits (for evaluation of CHF) and medication prescriptions, without structured follow-up or service of specialized nurses [9]. Patients in the telemonitoring groups received telemonitoring-guided CHF intervention in addition to usual care for 6 months post discharge. The 6-month duration is most commonly used for examination of the telemonitoring-guided intervention in clinical trials [10]. The telemonitoring-guided intervention included a telemonitoring system (installed at patients’ home) usually with a digital tablet as a central structural element to transmit vital elements from patients’ home to the hospital. Algorithms were programmed and implemented in this system to guide patient management (concomitant medication change, initiation of an ambulatory assessment by a home physician, or to hospitalize the patient). The patients in the telemonitoring groups might or might not adhere to the telemonitoring-guided management. If patients were not readmitted to the hospital during the 6-month telemonitoring-guided management period, they would be followed up by usual care only in the following cycles until rehospitalization occurred. Those who survived the next hospitalization would receive usual care alone or usual care plus 6-month telemonitoring-guided management according to patients’ NYHA classification and the corresponding study group.

### Clinical Inputs

The clinical parameters are listed in Multimedia Appendix 1. Literature search on MEDLINE over the period 2000 to 2019 was conducted using the keywords *heart failure*, *telemonitoring*, *remote patient monitoring*, *telehealth*, *telemedicine*, *telemedical center*, *hospitalization*, *admission*, and *mortality*. The inclusion criteria for clinical trials of CHF management were (1) reports written in English, (2) patients aged 18 years or older, and (3) the incidence of hospitalization or mortality was reported. A study was included if the data relevant to the model inputs were available. Case studies were excluded. Preferred studies were meta-analyses or randomized controlled trials. If multiple sources were found for a model input, the weighted average was used as the base case value and the high or low values formed as the range for sensitivity analysis.

The distribution of the hypothetical cohort of recently discharged patients with CHF among NYHA classes (upon entry to the present model) was estimated from the findings of the TIM-HF2 trial that the percentages of NYHA classes I, II, III, and IV in 1538 patients with CHF were 0.7%, 51.8%, 47.2%, and 0.3%, respectively [5]. The CHF-related hospitalization rate of patients with NYHA class I and the hazard ratios of hospitalization for patients with NYHA classes II, III, and IV (vs NYHA class I) were reported by the DIG trial, which demonstrated higher NYHA classification to be associated with increased risk of hospitalization. The hazard ratios of hospitalization for patients with NYHA classes II, III, and IV were 1.16 (95% CI 0.76-1.77; *P*=.50), 2.27 (95% CI 1.45-3.56; *P*<.001), and 3.71 (95% CI 1.25-11.02; *P*=.02), respectively [8]. The 3-year hospitalization rate of NYHA class I patients was 14.3% [8], and it was converted into a 6-month probability of 2.36% by the equation [11] p=1−e^−rt^ (p=probability, r=event rate, and t=cycle length). In the present model, the probabilities of hospitalization for patients with NYHA classes II, III, and IV were estimated by multiplying the probability of hospitalization for NYHA class I with the corresponding hazard ratios of hospitalization. A retrospective outcome study, including 2176 patients with heart failure, reported the hazard ratio of readmission for patients with prior admission to be 1.25 (95% CI 1.05-1.48; *P*=.01) [12]. We, therefore, estimated the readmission rate of patients in each NYHA class using the hazard ratio of readmission for patients with prior hospitalization and corresponding hospitalization rate of each NYHA class.

A network meta-analysis on telemonitoring interventions for heart failure patients included 13 randomized clinical trials with total 10,913 patients [10]. The telemonitoring with transmission of physiologic measurements (weight, blood pressure, and heart rate) was found to reduce CHF-related hospitalization (odds ratio 0.64, 95% CI 0.39-0.95) when compared with usual care alone, and these findings were adopted as the model input for the odds of hospitalization during telemonitoring-guided management versus usual care alone.

The 6-month probabilities of mortality in patients with CHF receiving usual care (0.65%, 3.56%, 11.68%, and 11.68% for NYHA classes I, II, III, and IV, respectively) were estimated by the yearly all-cause mortality rate of each NYHA class, retrieved from the outcomes of the control group in a clinical trial of 2737 patients with heart failure with 4783 patient-years of follow-up [13,14]. The probabilities of all-cause mortality in the telemonitoring groups were approximated by the probabilities of mortality in the usual care group and hazard ratio of mortality with telemonitoring versus usual care (0.70; 95% CI 0.50-0.96; *P*=.03, reported by the TIM-HF2 trial) [5]. Adherence rate (81%) for the telemonitoring-guided CHF management was estimated from the findings of the Telemedical Interventional Monitoring in Heart Failure trial [15]. In this trial, 81% of the study patients (n=354) in the telemonitoring arm achieved at least 70% of daily data transfer. In this model, we therefore adopted 70% as the lower threshold of daily data transfer for telemonitoring, and adherence rate of 81% (achieving lower threshold of daily data transfer) was used as the base case value. The hospitalization and mortality rates in the usual care group were applied to patients in the telemonitoring groups who did not achieve the lower threshold of data transfer.

The annual transition probabilities between NYHA classes were also retrieved from a prior clinical trial on patients with heart failure [13,14] and were converted to 6-month probabilities by the eigen decomposition approach using MATLAB (MathWorks) [16]. A previous cost-effectiveness analysis of patients with systolic heart failure assumed the same transition probabilities from NYHA class IV to classes I to III as of NYHA class III to three other classes, and the present model had adopted similar assumption [14]. Transition between NYHA classes was assumed to be the same for the telemonitoring and usual care groups in this study because of paucity of evidence to indicate influence of telemonitoring-guided management (if any) on the changes of NYHA classes.

### Utility Inputs

The QALY gain expected by each subject in the model was estimated from subject-time spent in various health statuses and the corresponding utility values. The utility inputs are shown in Multimedia Appendix 1. The base case utility and the decrement utility of CHF-related admission were retrieved from the findings of the standard care group in a randomized controlled trial, including 6505 patients with prior hospitalization for heart failure within 12 months [17]. The duration of hospitalization was approximated from the average total days per discharge in the diagnosis-related groups (DRGs) of heart failure reported by the Centers for Medicare and Medicaid Services [18]. The expected lifelong QALYs were discounted to the year 2019 with an annual rate of 3%.

### Cost Inputs

The health economic analysis was performed from the perspective of US health care providers, and the cost inputs were retrieved from the literature review and public data. The CHF inpatient cost was retrieved from the 2016 DRGs data [18]. The costs of hospitalization for NYHA classes I, II, and III to IV (including death occurred during hospitalization) were approximated by the charges per discharge for heart failure without complication, with complication, and with major complication, respectively. The monthly outpatient costs in different NYHA classes were estimated from findings of a resource utilization study of 117,870 patients with CHF in 2010 and the relative difference in inpatient costs between NYHA classes [18,19]. The monthly telemonitoring cost per patient was estimated by the total annual expense on home telehealth and the number of patients served reported by the Department of Veterans Affairs in 2018 [20,21]. The utilization of telemonitoring-guided management in patients who were nonadherence or died during telemonitoring treatment were both assumed to be 3 months (and examined over a range of 1-6 months in sensitivity analysis) for cost estimation. All cost inputs and the expected lifelong cost were discounted to the year 2019 with an annual rate of 3%.

### Cost-Effectiveness Analysis

Expected direct medical cost and QALYs gain were calculated for each strategy. The cost per QALY saved by each strategy versus universal usual care (the common baseline) was reported. A strategy was dominated by the comparator when it gained fewer QALYs at higher cost, and the dominated strategy was eliminated from further cost-effectiveness analysis. If a strategy gained additional QALYs at higher cost than the comparator, the incremental cost per QALY saved (ICER) of the more effective strategy was calculated using the following equation: △cost/△QALYs. The willingness-to-pay (WTP) threshold of US $50,000 per QALY was adopted in this analysis [22]. The study group gained the highest QALYs with ICER less than US$ 50,000 per QALY. This was considered as the preferred cost-effective option.

### Sensitivity Analysis

Sensitivity analysis was conducted to examine the robustness of the model results. One-way sensitivity analysis was conducted over the range specified in Multimedia Appendix 1. To evaluate the impact of all variables simultaneously, probabilistic sensitivity analysis was performed with 10,000 Monte Carlo simulations by randomly drawing each of the model inputs from the specific probability distribution indicated in Multimedia Appendix 1. The probability of each study arm to be accepted as the preferred option was determined over a wide range of WTP from US $0 to US $100,000 per QALY in the acceptability curve.

## Results

### Base Case Analysis

Base case results are shown in Table 1. All telemonitoring groups incurred higher QALYs at higher costs when compared with the universal usual care group. The universal telemonitoring group gained the highest QALYs (6.2967 QALYs), followed by the telemonitoring for class II to IV group (6.2960 QALYs), the telemonitoring for class III to IV group (6.2450 QALYs), and the universal usual care group (6.1530 QALYs).

When compared with universal usual care (the common baseline), the cost per QALY saved by each telemonitoring group ranged between US $35,393 and US $36,720 per QALY and was lower than the WTP threshold (US $50,000 per QALY). Comparing with the next less costly option (in an ascending order), the ICERs of the telemonitoring for class III to IV group (US $35,393 per QALY) and the telemonitoring for class II to IV group (US $38,261 per QALY) were lower than the WTP threshold, and the ICER of the universal telemonitoring group (US $100,458 per QALY) exceeded the WTP threshold. The telemonitoring for class II to IV group gained the highest QALYs with ICER less than the WTP threshold and was therefore the preferred cost-effective option.

**Table 1:** Base case results.

Treatment option	Direct medical cost (US $)	Incremental cost (US $)^a^	Incremental cost (US $)^b^	QALYs^c^	Incremental QALYs^a^	Incremental QALYs^b^	ICER^d^ (US $ per QALY)	Cost per QALY saved (US $ per QALY)^b^
Universal usual care	238,146	N/A^e^	N/A	6.1530	N/A	N/A	N/A	N/A
TM^f^ for class III to IV	241,401	3256	3256	6.2450	0.0920	0.0920	35,393	35,393
TM for class II to IV	243,354	1953	5209	6.2960	0.0510	0.1430	38,261	36,416
Universal TM	243,423	68	5277	6.2967	0.0007	0.1437	100,458	36,720

^a^ICER=(Total cost_TM_−Total cost_next less costly option_)/(QALY saved_TM_−QALY saved_next less costly option_).

^b^Cost per QALY saved=(Total cost_TM_−Total cost_universal UC_)/(QALY saved_TM_−QALY saved _universal UC_).

^c^QALYs: quality-adjusted life years.

^d^ICER: incremental cost-effectiveness ratio.

^e^N/A: not applicable.

^f^TM: telemonitoring.

### Sensitivity Analysis

#### One-Way Sensitivity Analysis

**Figure 2:**
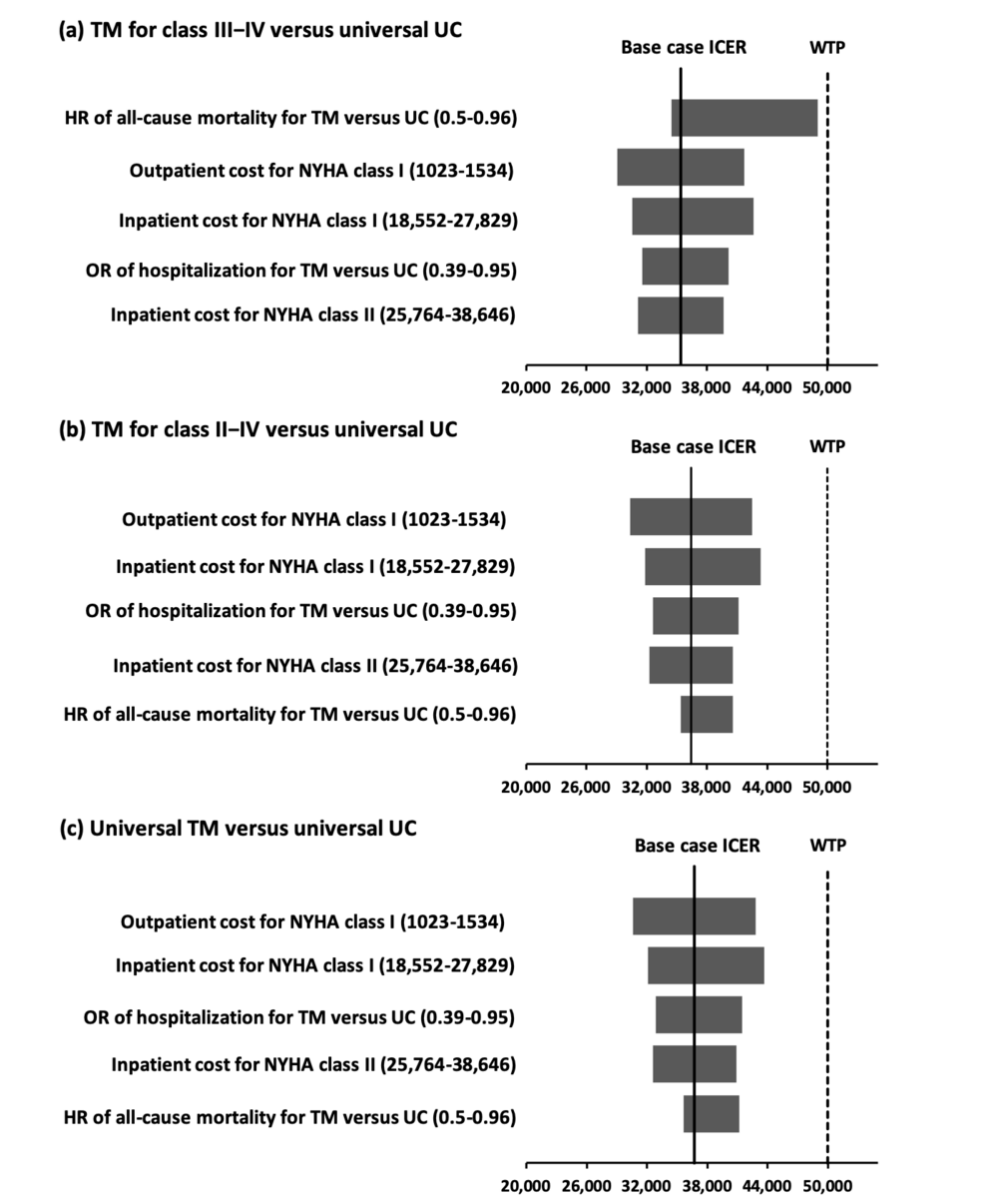
Tornado diagrams: (A) TM for class III to IV versus universal UC, (B) TM for class II to IV versus universal UC, (C) universal TM versus universal UC. HR: hazard ratio; ICER: incremental cost-effectiveness ratio; NYHA: New York Heart Association; OR: odds ratio; TM: telemonitoring; UC: usual care; WTP: willingness-to-pay.

The ICER of each telemonitoring strategy, compared with universal usual care, was examined by one-way sensitivity analysis. No threshold value was identified in the one-way sensitivity analysis. Five critical parameters with impact on the ICER of each telemonitoring strategy by 10% or greater were identified (Figure 2). Two of the critical parameters were clinical inputs: odds ratio of hospitalization for telemonitoring versus usual care and hazard ratio of all-cause mortality for telemonitoring versus usual care. The remaining three critical parameters were cost inputs: CHF hospitalization cost and monthly outpatient costs for NYHA class I and CHF hospitalization cost for NYHA class II.

To further explore the impact of adherence of telemonitoring-guided management and monthly telemonitoring cost, extended one-way sensitivity analyses were conducted over the lower limits of these variables (Multimedia Appendix 2). When the adherence to achieve lower threshold of data transfer was 20.9% to 30.8%, the telemonitoring for class III to IV group became the preferred option. Universal usual care was the preferred option if the patient adherence declined to less than 20.9%. When monthly telemonitoring cost reduced from US $193 (base case value) to less than US $87.5, universal telemonitoring would become the preferred strategy. The findings of the extended one-way sensitivity analysis are shown in Multimedia Appendix 2.

#### Probabilistic Sensitivity Analysis

**Figure 3:**
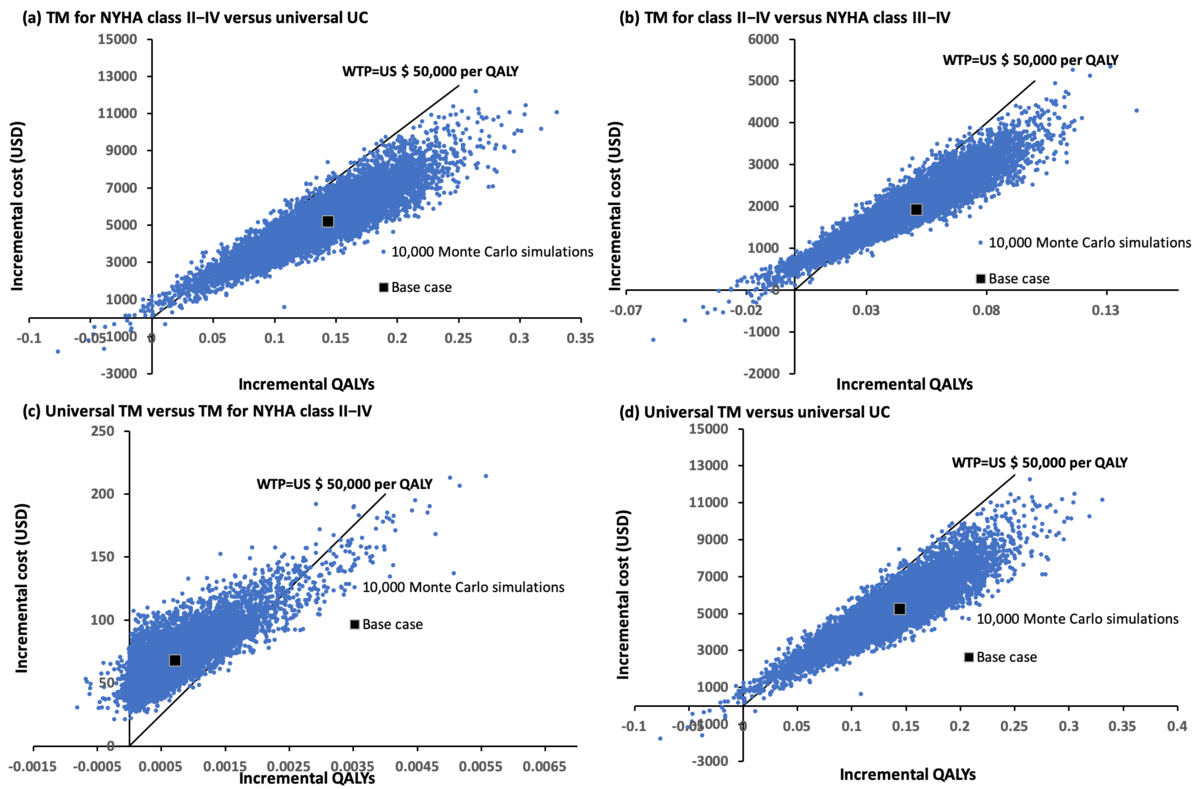
Scatter plots: (A) TM for class II to IV versus universal UC, (B) TM for class II to IV versus TM for class III to IV, (C) universal TM versus TM for class II to IV, (D) universal TM versus universal UC. QALY: quality-adjusted life year; TM: telemonitoring; UC: usual care; WTP: willingness-to-pay.

The probabilistic sensitivity analysis was performed by the 10,000 Monte Carlo simulations (Figure 3). Compared with the universal usual care group, the telemonitoring for class II to IV group gained a mean QALY of 0.1343 (95% CI 0.1334-0.1352; *P*<.001) with an additional mean cost of US $5062 (95% CI US $5031-US $5092; *P*<.001). Of the 10,000 simulations, the ICERs of the telemonitoring for class II to IV group were below the WTP threshold in 95.91% of time. Compared with the telemonitoring for class III to IV group, the telemonitoring for class II to IV group was more costly by US $2045 (95% CI US $2032-US $2058; *P*<.001) and gained 0.0490 QALYs (95% CI 0.0486-0.0494; *P*<.001). The telemonitoring for class II to IV group had ICERs (for additional QALYs gain) below WTP in 79.86% of the simulations. Compared with the telemonitoring for NYHA class II to IV group, the universal telemonitoring group was costlier by US $68 (95% CI US $68-US $69; *P*<.001) and gained 0.00064 QALYs (95% CI 0.00063-0.00065; *P*<.001). The ICERs of the universal telemonitoring group (vs telemonitoring for class II-IV group) were below the WTP in 3.30% of time.

As the ICER of universal telemonitoring versus the universal usual care group (US $36,720 per QALY) was below the WTP threshold in the base case analysis, a probabilistic sensitivity analysis was further conducted. The universal telemonitoring group incurred higher cost of US $5130 (95% CI 5099-5161; *P*<.001) and gained 0.1349 QALYs (95% CI 0.1340-0.1358; *P*<.001). The ICERs (for additional QALYs gained) of the universal telemonitoring group were below the WTP threshold in 95.52% of 10,000 simulations.

**Figure 4:**
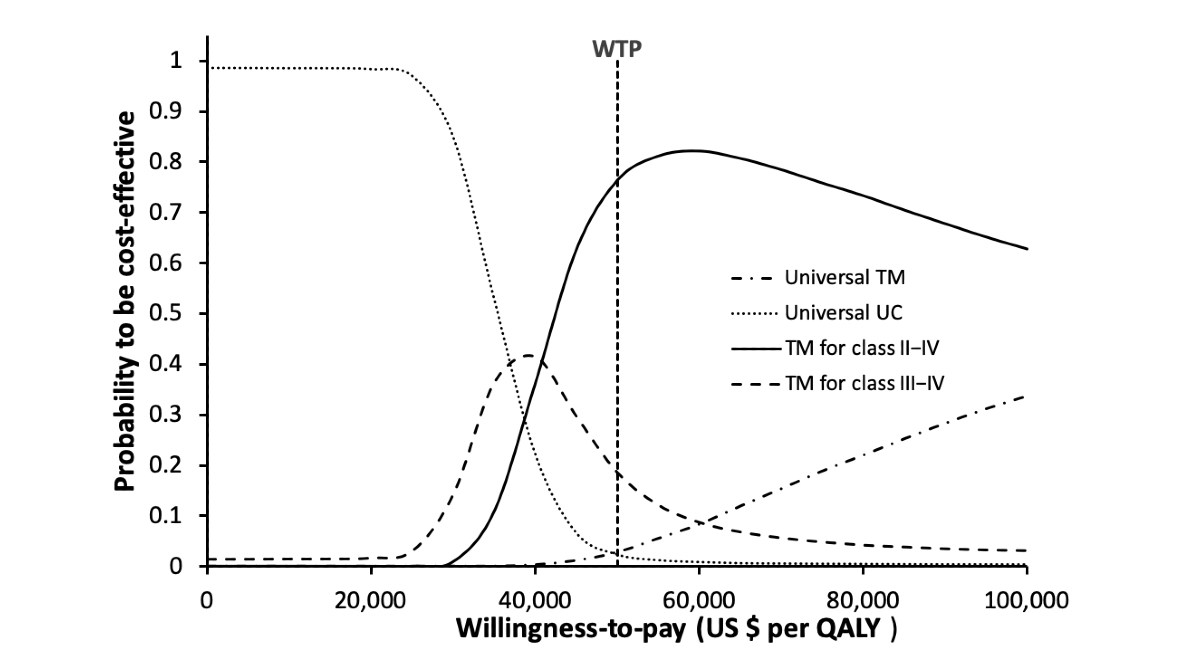
Cost-effectiveness acceptability curve for each strategy to be preferred option against the willingness-to-pay threshold. QALY: quality-adjusted life year; TM: telemonitoring; UC: usual care; WTP: willingness-to-pay.

The probability of each study group to be accepted as cost-effective was examined in the acceptability curves over a wide range of WTP (US $0-US $100,000 per QALY; Figure 4). At WTP of US $50,000 per QALY, the probabilities of the universal telemonitoring, telemonitoring for class II to IV, telemonitoring for class III to IV, and universal usual care groups to be accepted as the preferred option were 2.76%, 76.31%, 18.6%, and 2.33%, respectively.

## Discussion

### Principal Findings

The present model examined the potential clinical and economic outcomes of providing telemonitoring-guided CHF management with usual outpatient care to postdischarge patients with different NYHA classifications. Our findings showed that all telemonitoring plus usual care strategies versus usual care alone were cost-effective using US $50,000 per QALY as the WTP threshold from the perspective of US health care providers. Further comparison between telemonitoring groups showed that universal telemonitoring was more effective than telemonitoring for NYHA class II to IV in QALY gain, yet the ICER (US $100,458 per QALY) exceeded the WTP threshold. Telemonitoring for NYHA class II to IV was the most effective strategy with a WTP-acceptable ICER in the base case analysis. The robustness of base case findings was supported by the one-way analysis that no influential factor with threshold value was identified. The probabilistic sensitivity analysis further demonstrated that the strategy of providing telemonitoring for NYHA class II to IV had the highest probabilities to be accepted as cost-effective at the WTP threshold of US $40,830 to US $100,000 per QALY, as indicated by the acceptability curves.

### Limitations

This study was limited by the uncertainties on data availability and model assumptions. Rigorous sensitivity analyses were therefore performed to examine the impact of model input uncertainties on the robustness of the model results. The US health care costs were used as model inputs, potentially limiting the findings’ generalizability in health care systems of other countries. To enhance the transferability of the decision model to other countries, acquisition of country- and region-specific health care cost as model inputs is necessary. Indirect cost (eg, cost of caregivers and loss of productivity) was not included and might, therefore, underestimate the health economic impact of telemonitoring. A cost-effectiveness analysis from the perspective of society to include both direct and indirect costs on telemonitoring-guided management in patients discharged for CHF is highly warranted.

### Comparison With Prior Work

This study was the first cost-effectiveness analysis that examined the cost and QALY gained by telemonitoring-guided management for patients with CHF from the perspective of US health care providers. Previously, a cost-consequence analysis conducted from the perspective of US public payers over a 20-year time horizon found the telehealth program for CHF management to be likely to save cost (from US $3422 to US $4456) and gain 0.46 to 0.50 life-years for high-risk (including prior hospitalization) patients [23]. The findings of this prior analysis supported our results that telemonitoring-guided management was effective in saving life-years and QALYs. The incremental cost incurred to telemonitoring groups in this analysis was attributed to the higher current (year 2019) costs of CHF care in both inpatient and outpatient settings, whereas the cost saving reported in the prior US analysis was generated by lower CHF inpatient and outpatient costs (originated in the year 2010 and earlier). A cost-effectiveness analysis on telemonitoring, usual care, and nurse telephone support for CHF patients was performed from the perspective of a third-party payer of the Netherlands [24]. A Markov model was used to examine the disease progression over four classes of NYHA classifications (I-IV) and death in the time horizon of 20 years. The analysis reported that the telemonitoring group gained higher QALYs at an additional cost than the usual care group. Our findings were consistent with the Netherlands study, and we further examined the impact of two factors, which were less transferable from region to region: patient adherence to telemonitoring and monthly telemonitoring cost on the cost-effective acceptance of telemonitoring-guided management in various levels of CHF severity.

In the Telemonitoring to Improve Heart Failure Outcomes and Better Effectiveness After Transition–Heart Failure studies, the adherence to telemonitoring decreased by nearly half within the first 30 days [25,26]. A threshold of 20% to 30% of patients to adhere to 70% of data transfer was identified in the present model. For a health care system in which patients’ adherence to 70% or greater daily data transfer for telemonitoring is less than 20%, the telemonitoring-guided service might not be acceptable as a cost-effective option for all patients with CHF. If the adherence ranged between 20.9% and 30.8%, telemonitoring-guided management for the patients with more severe CHF (NYHA class III-IV) was likely to be cost-effective. If the adherence was higher than 30.8%, telemonitoring-guided management would likely be the preferred cost-effective option for patients in NYHA class II and above. The adherence of patients varies among different health care settings, and the collection of local adherence data is therefore critical to inform the decision-making process of the health care providers on the implementation of telemonitoring-guided service.

With the advancement of digital technology, the costs of devices and the computational techniques applied in the telemonitoring-guided management are anticipated to decrease over time. If the cost of telemonitoring-guided interventions is decreased to US $87.5 per patient per month or less (as indicated by the extended sensitivity analysis), the universal telemonitoring-guided management would be acceptable as the preferred option for all patients with CHF from the perspective of health service providers.

### Conclusions

In conclusion, universal usual outpatient care for all discharged patients with CHF plus telemonitoring-guided management for those with NYHA class II to IV appears to be the preferred cost-effective strategy from the perspective of US health care providers.

## Appendix

Multimedia Appendix 1 Model inputs.

Multimedia Appendix 2 Results of extended one-way sensitivity analysis.

## Abbreviations

CHF: chronic heart failure
DIG: Digitalis Investigation Group
DRG: diagnosis-related group
ICER: incremental cost-effectiveness ratio
NYHA: New York Heart Association
QALY: quality-adjusted life year
TIM-HF2: Efficacy of Telemedical Interventional Management in Patients with Heart Failure II
WTP: willingness-to-pay
