# Supplementary material for: Telemonitoring Versus Usual Care for Elderly Patients With Heart Failure Discharged From the Hospital in the United States: Cost-Effectiveness Analysis
Source: JMIR Mhealth Uhealth. 2020 Jul 6;8(7):e17846. doi: 10.2196/17846 (PMC7381019; doi:10.2196/17846)
Supplement: Multimedia Appendix 1 [file mhealth_v8i7e17846_app1.docx]

**Table S1.** Model inputs.

| Parameters | | | Base case value | Range for sensitivity analysis | Distribution | Reference |
| --- | --- | --- | --- | --- | --- | --- |
| **Clinical inputs** | | | | | | |
|  | **Proportion of NYHA^a^ classification (%)** | | | | Dirichlet | 1 |
|  |  | NYHA class I | 0.7 | 0.4-1 |  |  |
|  |  | NYHA class II | 51.8 | 51-52 |  |  |
|  |  | NYHA class III | 47.2 | 46.9-47.5 |  |  |
|  |  | NYHA class IV | 0.3 | 0.26-0.39 |  |  |
|  | **Transition probabilities** | | | | Dirichlet | 2,3 |
|  |  | I to I | 0.7872 | 0.6298-0.9446 |  |  |
|  |  | I to II | 0.2100 | 0.1680-0.2520 |  |  |
|  |  | I to III | 0.0028 | 0.0022-0.0034 |  |  |
|  |  | I to IV | 0 | 0-0.0034 |  |  |
|  |  | II to I | 0.0383 | 0.0306-0.0460 |  |  |
|  |  | II to II | 0.9356 | 0.0187-0.0281 |  |  |
|  |  | II to III | 0.0234 | 0.0187-0.0281 |  |  |
|  |  | II to IV | 0.0027 | 0.0022-0.0050 |  |  |
|  |  | III to I | 0.0088 | 0.007-0.01060 |  |  |
|  |  | III to II | 0.2308 | 0.1846-0.2770 |  |  |
|  |  | III to III | 0.7022 | 0.5618-0.8426 |  |  |
|  |  | III to IV | 0.0582 | 0.0466-0.0700 |  |  |
|  |  | VI to I | 0.0088 | 0.007-0.01060 |  |  |
|  |  | VI to II | 0.2308 | 0.1846-0.2770 |  |  |
|  |  | VI to III | 0.7022 | 0.5618-0.8426 |  |  |
|  |  | VI to IV | 0.0582 | 0.0466-0.0700 |  |  |
|  | **All-cause mortality of UC^b^ (%)** | | | | Beta | 2,3 |
|  |  | NYHA class I | 0.65 | 0.52-0.78 |  |  |
|  |  | NYHA class II | 3.56 | 2.85-4.27 |  |  |
|  |  | NYHA class III | 11.68 | 9.34-14.02 |  |  |
|  |  | NYHA class IV | 11.68 | 9.34%-14.02 |  |  |
|  | Hazard ratio of all-cause mortality (TM^c^ vs UC) | | 0.70 | 0.50-0.96 | Lognormal | 1 |
|  | Probability of hospitalization for NYHA class I in UC | | 2.36 | 1.88-2.83 | Beta | 4 |
|  | **Hazard ratio of hospitalization (vs NYHA class I)** | | | | Lognormal | 4 |
|  |  | NYHA class II | 1.16 | 0.76-1.77 |  |  |
|  |  | NYHA class III | 2.27 | 1.45-3.56 |  |  |
|  |  | NYHA class IV | 3.71 | 1.25-11.02 |  |  |
|  | Hazard ratio of readmission (prior rehospitalization vs no prior hospitalization) | | 1.25 | 1.05-1.48 | Lognormal | 5 |
|  | Odds ratio of hospitalization (TM vs UC) | | 0.64 | 0.39-0.95 | Lognormal | 6 |
|  | Adherence to TM-guided management (%) | | 81 | 65-97 | Beta | 7 |
| **Utility inputs** | | | | | | |
|  | **Baseline utilities** | | | | Uniform | 8 |
|  |  | NYHA class I | 0.82 | 0.78-0.85 |  |  |
|  |  | NYHA class II | 0.74 | 0.69-0.75 |  |  |
|  |  | NYHA class III | 0.64 | 0.55-0.77 |  |  |
|  |  | NYHA class IV | 0.46 | 0.41-0.61 |  |  |
|  | **Utility decrement because of hospitalization** | | | | Uniform | 8 |
|  |  | NYHA class I | 0.04 | 0.03-0.05 |  |  |
|  |  | NYHA class II | 0.07 | 0.06-0.08 |  |  |
|  |  | NYHA class III | 0.10 | 0.08-0.12 |  |  |
|  |  | NYHA class IV | 0.29 | 0.23-0.35 |  |  |
|  | **Length of hospitalization (days)** | | | | Triangular | 9 |
|  |  | NYHA class I | 6 | 5-7 |  |  |
|  |  | NYHA class II | 4 | 3-5 |  |  |
|  |  | NYHA class III | 3 | 2-4 |  |  |
|  |  | NYHA class IV | 3 | 2-4 |  |  |
| **Cost inputs** | | | | | | |
|  | Monthly cost of TM (US $) | | 193 | 150-230 | Triangular | 10,11 |
|  | Duration of TM for nonadherent patients (months) | | 3 | 1-6 | Triangular | Assumption |
|  | Duration of TM for patients dead (months) | | 3 | 1-6 | Triangular | Assumption |
|  | **Hospitalization (per episode) (US $)** | | | | Triangular | 9 |
|  |  | NYHA class I | 23,191 | 18,552-27,829 |  |  |
|  |  | NYHA class II | 32,205 | 25,764-38,646 |  |  |
|  |  | NYHA class III | 48,691 | 38,953-58,429 |  |  |
|  |  | NYHA class IV | 48,691 | 38,953-58,429 |  |  |
|  | **Outpatient cost** **(US $)** | | | | Triangular | 9,12 |
|  |  | NYHA class I | 1279 | 1023-1534 |  |  |
|  |  | NYHA class II | 1776 | 1421-2131 |  |  |
|  |  | NYHA class III | 2685 | 2148-3222 |  |  |
|  |  | NYHA class IV | 2685 | 2148-3222 |  |  |
|  | All-cause death (US $) | | 48,691 | 38,953-58,429 | Triangular | 9 |

^a^NYHA: New York Heart Association.

^b^UC: usual care.

^c^TM: telemonitoring.

References:

1. Koehler F, Koehler K, Deckwart O, et al. Efficacy of telemedical interventional management in patients with heart failure (TIM-HF2): a randomised, controlled, parallel-group, unmasked trial. Lancet 2018;392(10152):1047-1057. PubMed PMID: 30153985.
2. Ademi Z, Pasupathi K, Liew D. Cost-Effectiveness of Eplerenone Compared to Usual Care in Patients With Chronic Heart Failure and NYHA Class II Symptoms, an Australian Perspective. Medicine (Baltimore) 2016;95(18):e3531. PubMed PMID: 27149456.
3. Zannad F, McMurray JJ, Krum H, et al. Eplerenone in patients with systolic heart failure and mild symptoms. N Engl J Med 2011;364(1):11-21. PubMed PMID: 21073363.
4. Ahmed A, Aronow WS, Fleg JL. Higher New York Heart Association classes and increased mortality and hospitalization in patients with heart failure and preserved left ventricular function. Am Heart J;151(2):444-450. PubMe PMID: 16442912.
5. Krumholz HM, Chen YT, Wang Y, Vaccarino V, Radford MJ, Horwitz RI. Predictors of readmission among elderly survivors of admission with heart failure. Am Heart J 2000;139(1):72-77. PubMed PMID: 10618565.
6. Kotb A, Cameron C, Hsieh S, Wells G. Comparative effectiveness of different forms of telemedicine for individuals with heart failure (HF): a systematic review and network meta-analysis. PLoS One 2015;10(2):e0118681. PubMed PMID: 25714962.
7. Koehler F, Winkler S, Schieber M, et al. Impact of remote telemedical management on mortality and hospitalizations in ambulatory patients with chronic heart failure: the telemedical interventional monitoring in heart failure study. Circulation 2011;123(17):1873-1880. PubMed PMID: 21444883.
8. Griffiths A, Paracha N, Davies A, Branscombe N, Cowie MR, Sculpher M. Analyzing Health-Related Quality of Life Data to Estimate Parameters for Cost-Effectiveness Models: An Example Using Longitudinal EQ-5D Data from the SHIFT Randomized Controlled Trial. Adv Ther 2017;34(3):753-764. PubMed PMID: 28205056.
9. Center for Medicare and Medicaid services. 100% MEDPAR Inpatient Hospital Data for Fiscal Year 2016. URL: https://www.cms.gov/Research-Statistics-Data-and-Systems/Statistics-Trends-and-Reports/MedicareFeeforSvcPartsAB/Downloads/DRGState16.pdf. [accessed September 15, 2019].
10. Department of Veterans Affairs. Volume II Medical Programs and Information Technology Programs. URL: https://www.va.gov/budget/docs/summary/fy2020VAbudgetVolumeIImedicalProgramsAndInformationTechnology.pdf. [accessed September 28, 2019].
11. Congressional Research Service. Department of Veterans Affairs (VA): A Primer on Telehealth. URL: https://fas.org/sgp/crs/misc/R45834.pdf. [accessed September 29, 2019].
12. Yoon J, Fonarow GC, Groeneveld PW, et al. Patient and Facility Variation in Costs of VA Heart Failure Patients. JACC Heart Fail 2016;4(7):551-558. PubMed PMID: 26970829.
