# Supplementary material for: Telemonitoring Versus Usual Care for Elderly Patients With Heart Failure Discharged From the Hospital in the United States: Cost-Effectiveness Analysis
Source: JMIR Mhealth Uhealth. 2020 Jul 6;8(7):e17846. doi: 10.2196/17846 (PMC7381019; doi:10.2196/17846)

**Figure S1 (a) QALYs (b) cost of each strategy against percentage of patients who achieved adherence to TM-guided management. QALYs, quality-adjusted life-years; TM, telemonitoring; UC, usual care. Adherence level of 70% or above with daily data transfer was adopted as adherence.**


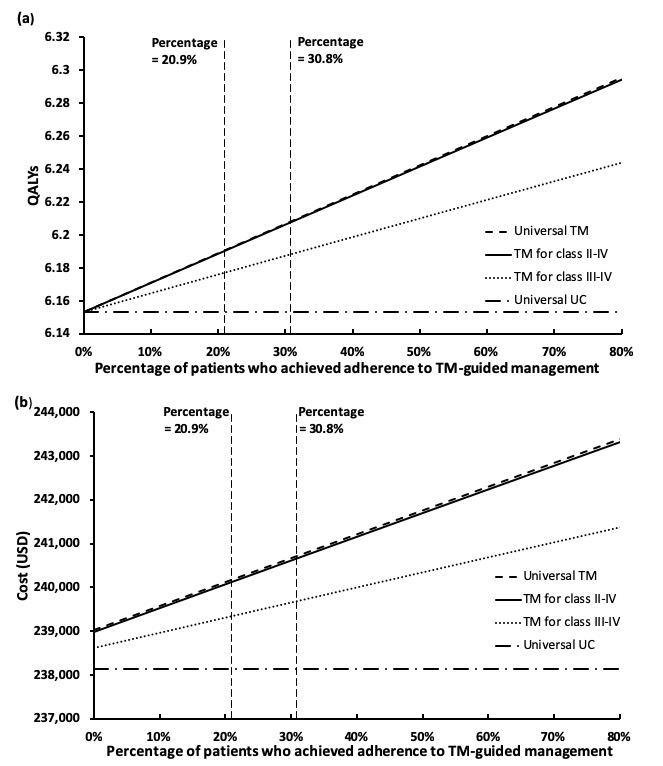


**Figure S2 (a) QALYs (b) cost of each strategy against monthly TM cost per patient. QALYs, quality-adjusted life-years; TM, telemonitoring; UC, usual care**


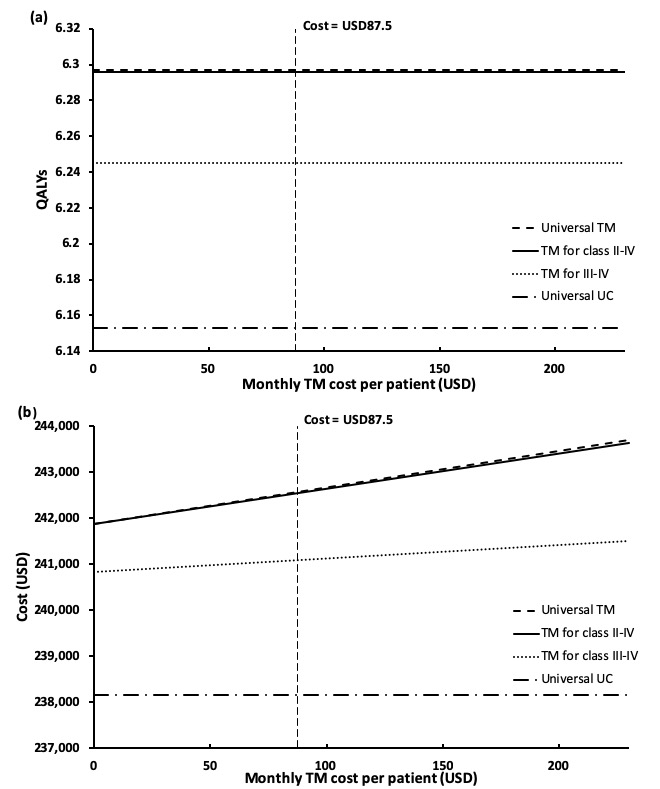

Supplement: Multimedia Appendix 2 [file mhealth_v8i7e17846_app2.docx]
